# Genome Wide Association Studies (GWAS) Identify QTL on SSC2 and SSC17 Affecting Loin Peak Shear Force in Crossbred Commercial Pigs

**DOI:** 10.1371/journal.pone.0145082

**Published:** 2016-02-22

**Authors:** Chunyan Zhang, Heather Bruce, Tianfu Yang, Patrick Charagu, Robert Alan Kemp, Nicholas Boddicker, Younes Miar, Zhiquan Wang, Graham Plastow

**Affiliations:** 1 Department of Agricultural, Food and Nutritional Sciences, University of Alberta, Edmonton, AB, Canada; 2 Hypor Inc., Regina, SK, Canada; 3 Genesus Inc., Oakville, MB, Canada; China Agricultural Univeristy, CHINA

## Abstract

Of all the meat quality traits, tenderness is considered the most important with regard to eating quality and market value. In this study we have utilised genome wide association studies (GWAS) for peak shear force (PSF) of loin muscle as a measure of tenderness for 1,976 crossbred commercial pigs, genotyped for 42,721 informative SNPs using the Illumina PorcineSNP60 Beadchip. Four 1 Mb genomic regions, three on SSC2 (at 4 Mb, 5 Mb and 109 Mb) and one on SSC17 (at 20 Mb), were detected which collectively explained about 15.30% and 3.07% of the total genetic and phenotypic variance for PSF respectively. Markers ASGA0008566, ASGA0008695, DRGA0003285 and ASGA0075615 in the four regions were strongly associated with the effects. Analysis of the reference genome sequence in the region with the most important SNPs for SSC2_5 identified *FRMD8*, *SLC25A45* and *LTBP3* as potential candidate genes for meat tenderness on the basis of functional annotation of these genes. The region SSC2_109 was close to a previously reported candidate gene *CAST*; however, the very weak LD between DRGA0003285 (the best marker representing region SSC2_109) and *CAST* indicated the potential for additional genes which are distinct from, or interact with, *CAST* to affect meat tenderness. Limited information of known genes in regions SSC2_109 and SSC17_20 restricts further analysis. Re-sequencing of these regions for informative animals may help to resolve the molecular architecture and identify new candidate genes and causative mutations affecting this trait. These findings contribute significantly to our knowledge of the genomic regions affecting pork shear force and will potentially lead to new insights into the molecular mechanisms regulating meat tenderness.

## Introduction

Eating quality of pork is mostly linked with wholesomeness, freshness, leanness, juiciness, taste and tenderness, with tenderness being considered the most important feature of eating quality and the primary factor influencing consumer purchase decisions [1–3]. For this reason, improving meat tenderness has become a high priority for the pork industry in order to better satisfy consumers`preferences for a reliable and pleasurable eating experience. This is important for the swine industry to maintain its sustainability and enhance its competiveness in terms of both swine genetics and sales, including export, of pork.

Shear force, as a moderately heritable trait (0.30), is a good indicator used to evaluate the degree of tenderness of pork [4]. The heritability of shear force in this dataset was even higher (0.39 ± 0.06) as reported recently [5]. Improving tenderness by traditional breeding methods is still a challenge due to the high cost of measurement and the limited amount of data available post-mortem. In recent decades, about 183 QTL have been mapped for pork shear force (PigQTLdb, release 27, August 2015. http://www.animalgenome.org/cgi-bin/QTLdb/SS/summary), but only a few gene variants, such as *CAST* (calpastatin) [6–13], have been shown to affect pork tenderness. With the development of high-density SNP chips and genome wide statistical analysis, GWAS have been increasingly used to identify more precise genomic regions and markers associated with quantitative traits, and the results have been incorporated into dairy cattle and poultry breeding programs. Both marker assisted selection and especially genomic prediction have been used, as reviewed by Goddard and Hayes [6]. Recently, more and more GWAS have been conducted for pork quality traits and several genomic regions have been identified [7,10–12,14]. However, only one of these reports was focused on pork tenderness and four SNPs were found to be significantly associated with slice shear force [14].

Therefore, the aims of this study were to detect genomic regions and identify potential candidate genes and markers affecting pork peak shear force in commercial crossbred pigs. The results could contribute useful information for marker based genomic improvement of meat tenderness.

## Materials and Methods

### Ethics Statement

The animals used in this study were raised as part of commercial pork production. The proposed work was reviewed by the University of Alberta Animal Care and Use Committee and considered as Category A (little of no animal manipulation) and no formal ethics approval was required. No other specific permissions were required for the work as the animals were produced as part of commercial pig breeding and pork operations and cared for according to the Canadian Quality Assurance Program, see http://www.cqa-aqc.com/resources-materials-e.php, which includes attention to animal health and well-being and is in line with the Canadian Council on Animal Care [9] guidelines. The health of the animals was assessed daily and in the case of severe injury or when pigs failed to respond to treatment, they were humanely euthanized.

### Animals and phenotypic data

A total of 1,976 crossbred commercial pigs from two populations (994 samples from Hypor and 982 samples from Genesus) were obtained for GWAS. All animals were from a typical Canadian three-way cross consisting of a Duroc sire line crossed with an F1 female (Landrace * Large White). All individuals were fed *ad libitum* and sent to the same processing plant when body weight was close to 115 kg during years 2010–2012. Briefly, pigs were housed overnight at a Federally approved abattoir (East 40 Packers, Brandon, Manitoba, Canada) with *ad libitum* access to water. Animals were inspected upon arrival and before slaughter and the slaughter process (electrical stunning followed by exsanguination) adhered to Government of Canada Guidelines. Carcasses were processed within 24 h post-mortem and pork loins (4^th^ -7^th^ ribs) were harvested from each pig, vacuum packaged, and chilled (2–4°C) within 24 hours after exsanguination. All the packaged loins in the same batch were simultaneously frozen (-20°C) within 24 hours after exsanguination and maintained frozen until the thawed muscle meat quality measurement. Prior to testing, the pork loins were thawed for 72 hours at 4°C. All samples were treated in the same way.

A final roast section from the loin approximately 8 cm thick was trimmed of exterior fat and connective tissue into a rectangle weighing approximately 250 g. The samples were cooked in a Whirl-pak bag partially submerged in a 73°C water bath to a final temperature of 71°C, as recorded by a Tinytag temperature probe (Thermistor probe TV-4020 Gemini Data Loggers Ltd., West Sussex, UK). Peak shear force (PSF) (N) was measured on six 1 cm^2^ rectangular cores cut from the roast that had been refrigerated overnight at 4°C. PSF was evaluated at a shear speed of 200 mm/min using a material testing machine (Lloyd Instruments Ltd., Surrey, UK) fitted with a Warner-Bratzler-like blade. Data from six rectangular cores were recorded using the NEXYGEN computer program (NEXYGEN Corporation, USA). The average from the six readings was taken as the final PSF value.

Other meat quality traits were also measured for further investigation, including drip loss (%), pH 24h post mortem, Minolta colour scores (L*, a* and b*) measured on fresh and frozen/thawed loin, and Minolta L*, a* and b* measured on different types of muscle of fresh ham. The details of these measurements have been described in our recent publication [5]. Miscellaneous muscles remaining on the rib after the loin eye muscle (m. *longissimus thoracis et lumborum*) was removed were trimmed from the vertebrae and rib bones, homogenized in a commercial food processor for crude fat content (%), crude protein content (%) and crude moisture content (%) evaluation using the FOSS FoodScan analyzer (FoodScan Lab, Type 78800, FOSS, Hillerød, Denmark). Duplicate analyses were performed on each sample and the average was used as the final value.

### Population stratification and linkage disequilibrium (LD) estimation

Population stratification was analyzed using an identical-by-state distance clustering method using the PLINK program [15]. The linkage disequilibrium (LD) of the pairwise SNPs was measured using r^2^ by *proc allele* using SAS 9.3. The haplotype block pattern was built using haploView software [16].

### SNP array genotyping and filtering

Genomic DNA was isolated from tissue samples following the DNA Extraction instruction manual (Thermo Fisher Scientific Ltd., Ottawa, ON, Canada). Genotyping was conducted by Delta Genomics (Edmonton, AB, Canada) using Illumina PorcineSNP60 V2 Genotyping Beadchip according to the Illumina Infinium Assay (Illumina, Inc., San Diego, CA, USA).

Quality control for genotypes was performed for the individuals with a call rate ≥ 95%. The SNPs were selected with the criteria of genotyping call rate ≥ 95%, *P_value* of chi-square test of Hardy-Weinberg equilibrium > 10^−6^, and minor allele frequency (MAF) ≥ 5%. After filtering, 1,976 individuals and 42,721 SNPs remained for the final GWAS analysis.

SNP positions were updated according to the newest release from Ensembl (Sscrofa 10.2 genome version). Map information was adjusted based on the LD decay for each chromosome. Only the SNPs with reliable positions were used in this study. The missing genotypes were imputed by Fimpute v2.2 [17].

A sub-population consisting of 831 individuals randomly selected from Genesus (430) and Hypor (401) was used to investigate the relationships of *CAST* and the detected QTL from GWAS. Three characteristic SNPs of *CAST* including *Ser66Asn*, *Ser638Arg* and 41642_408i reported by [18] [19] were included in an improved custom 96 SNP panel [13]. The DNA samples of the sub-population (n = 831) were genotyped with the custom 96 SNP panel as described previously [13].

### Phenotype adjustment

The original phenotypic records were adjusted within each population using the GLM procedure of SAS 9.3. A contemporary group effect was defined to include year and group during the test (~ 70–115 kg) and slaughter batch. There were 57 contemporary groups for Genesus and 46 for Hypor. Fixed effects of sex and contemporary group were fitted in the model below. For the Hypor population, the room and pen were fitted as fixed effects in the model as they were available and significant (*P < 0*.*05*) for the trait. For the Genesus population, all animals within a slaughter batch were raised in the same pen. Preliminary analyses indicated that the interactions among these factors were not significant (*P > 0*.*05*) and therefore ignored in the model.

$$y_{ijkl}=\mu +s_{i}+g_{j}+r_{k}+p_{l}+e_{ijkl}$$

Where *y*_*ijkl*_ is phenotypic observation, *μ* is overall mean, *s*_*i*_ is the effect of the *i*^*th*^ sex (1 = male and 2 = female), *g*_*j*_ is the effect of the *j*^*th*^ contemporary group (j = 1–57 for Genesus and 58–103 for Hypor), *r*_*k*_ is effect of the *k*^*th*^ room (k = 1–10 for Hypor), *p*_*l*_ is the effect of the *l*^*th*^ pen (l = 1–13 for Hypor), and *e*_*ijkl*_ is the random residual $\left[e_{ijkl}∼N(0,\sigma_{e}^{2})\right]$. The adjusted phenotype calculated as residual plus mean was used for the subsequent association studies.

### Genome wide association analyses

The adjusted phenotypes were used for the GWAS analysis, which were conducted in each (Hypor and Genesus) population separately and the combined data set. Population (Hypor and Genesus) was fitted as a fixed effect in the model for the combined data. The marker effects were estimated by fitting all SNPs simultaneously using Bayesian methods as implemented in the online software GenSel as follows:

$$y=\mathrm{Xb}+\sum_{j}^{k}{z_{j}\alpha }_{j}\delta_{j}+\varepsilon$$

Where, ***y*** = vector of adjusted phenotypic observations, ***X*** = incidence matrix relating fixed factors to phenotypes, ***b*** = design matrix of fixed effects (mean and population), ***z***_***j***_ = design matrix for the genotype covariate for SNP *j* (*j* = 1 to k) based on the copy number of the B alleles (***z***_***j***_ ***= 0*, *1 or 2)***, **α**_***j***_ = allele substitution effect for SNP *j*, and ***δ***_***j***_ = indicator for whether SNP *j* was included (***δ***_***j***_ = 1) or excluded (***δ***_***j***_ = 0) in the model for a given iteration of the Markov chain Monte Carlo procedure. A total of 61000 iterations were run with the first 1000 iterations discarded as burn-in. The probability of ***δ***_***i***_ = 0 was set equal to π = 0.99. The Bayesian model was implemented using method Bayes B. Genomic regions associated with the trait were identified using 1 Mb, non-overlapping windows using Ensembl build 10.2 of the swine genome.

The revised GWAS which included the additional SNPs of *CAST* genotyped with a small panel were conducted in a sub-population (n = 831). The method and statistical model were the same as described above.

### Further marker effect analysis

Further analysis of marker effect estimation and significance test was conducted in the combined data set. Regional genomic estimated breeding values (GEBV) for the detected 1 Mb windows were calculated as the sum of the marker effects of the SNPs within the region. The GEBV was regressed on single SNP variant (0/1/2) within the region as most of the SNPs within the region were in very weak LD. The R squared (RSQ) for the regression model, defined as the percentage of the regional GEBV variance explained by the SNP, was used to determine the most important SNP within that region affecting the trait.

The significant test and least square means (LSMs) for the interesting SNPs (both from GWAS and in *CAST*) were examined with a multi-marker association model implemented using ASReml [20]. The model equation was: *Y* = *Xb* + *Za* + *e*, where *Y* is the vector of adjusted phenotypes, *b* is the design matrix for fixed effects including mean, population and fitted markers, *a* is the random polygenic effect excluding the examined marker effects, which was assumed with $\left[u∼N(0,{A\sigma }_{\text{a}}^{2}\right]$, where ***A*** is the relationship matrix constructed based on pedigree information, and $\sigma_{\text{a}}^{2}$ is the polygenetic additive variance, *e* is the vector of residual errors with a distribution of $\left[e∼N(0,{I\sigma }_{e}^{2})\right]$, where ***I*** is the identity matrix and $\sigma_{e}^{2}$ is the residual variance. **X** and **Z** are the incidence matrices for b and *a*, respectively.

### Post-GWAS Bioinformatics analysis

Candidate gene identification and functional annotation for the interesting SNPs were obtained using Ensembl annotation of Sscrofa 10.2 genome version (http://uswest.ensembl.org/biomart). The functions and related information about the genes were summarized using the available database of GENECARDS (http://www.genecards.org/).

## Results

### Genomic regions

Population stratification showed that all animals from the two commercial populations could be classified into two clusters, which corresponded to the populations from which the individuals were derived. Therefore, GWAS were conducted in each population and the combined data set, respectively. In the combined data set, population was fitted as a fixed effect to optimize the association analysis model.

The top genomic regions in each data set were shown in Table 1. Two different regions on SSC2 were detected in the separate populations, 5 Mb and 27 Mb for population 1, and 106 Mb and 109 Mb for population 2. When the data sets were combined, four 1 Mb genomic regions, including SSC2_5 and SSC2_109, were found to have large effects on peak shear force Collectively, they explained about 15.30% of the total genetic variance and contributed about 3.07% of the total phenotypic variance. The details of these four regions were further analyzed as below.

**Table 1:** Genomic regions for loin peak shear force (PSF) detected by Bayes B analysis.

Population	Regions (SSC and position (Mb))	1^st^ SNP	Last SNP	No. of SNPs	% GenVar^a^	% PheVar^b^
Population 1	2_5	ASGA0008585	M1GA0002408	27	7.24	2.75
	2_27	MARC0035544	MARC0094546	37	1.36	0.52
Population 2	2_106	ALGA0116861	MARC0042944	16	3.04	0.77
	2_109	DRGA0003276	ASGA0011111	20	1.27	0.32
Combined data	2_4	ALGA0011480	ASGA0008575	11	2.26	0.45
	2_5	ASGA0008585	M1GA0002408	26	4.88	0.98
	2_109	DRGA0003276	ASGA0011111	17	6.53	1.31
	17_20	ALGA0093561	ASGA0075660	18	1.63	0.33

^a^: percentage of the total marker genetic variance explained by each region.

^b^: percentage of the total phenotypic variance explained by each region, which was calculated as the percentage of genetic variance (% GenVar) multiplied by molecular heritability (the percentage of the whole phenotypic variance explained by the fitted SNPs).

### Detailed analysis of QTL on SSC2

In combined data set, three 1 Mb regions on SSC2 associated with PSF were located at 4 Mb, 5 Mb and 109 Mb, respectively. A total of 54 SNPs were included and the LD (r^2^) relationships for these SNPs were very weak based on an overview of the haplotype LD block maps (S1, S2 and S3 Figs). The most important SNPs in each region were shown in Table 2. For region SSC2_4, the second SNP (SNP2) had a perfect RSQ (1.00) and was considered as the most important marker in this region in terms of an effect on the trait. For region SSC2_5, three SNPs (20, 25 and 26) with almost complete LD (r^2^ = 0.96–1.00) captured an average 91.8%, 98.7% and 95.5% of the variance for the estimated GEBV in the window, indicating that any of these SNPs could be affecting the trait. SNP25 was chosen for further analysis due to it having the highest RSQ (0.987). For region SSC2_109, the 11^th^ SNP (SNP11) captured more than 99% of the genetic variance and was considered to be the most important SNP for this region.

**Table 2:** The most important SNPs in the detected regions.

Regions	SNP_ID	SNP_Name	Position	MAF	RSQ^a^
2_4	SNP2	ASGA0008566	4161164	0.45	1.000
2_5	SNP20	ASGA0008662	5849903	0.40	0.918
	SNP25	ASGA0008695	5956519	0.40	0.987
	SNP26	M1GA0002408	5984348	0.40	0.955
2_109	SNP11	DRGA0003285	109670791	0.19	0.995
17_20	SNP7	ASGA0075615	20488772	0.21	0.966

^**a**^: The R squared (RSQ) of the window GEBV regressed on the SNP covariate (0/1/2), indicating the percentage of the GEBV variance explained by the SNP.

To date, calpastatin has been regarded as the most important candidate gene for pork tenderness. *CAST* was mapped to SSC2: 106934106–107128389 which is close to the third region (SSC2_109) found in the present study. Five SNPs in *CAST* were included in the Illumina PorcineSNP60 BeadChip. Two of them (ASGA0096522 and ASGA0011047), located in intron 3 and intron 30 of *CAST*, remained in the GWAS but did not show a significant effect on PSF at the genome wide level. The other three were discarded from the GWAS as one was uninformative and the other two (MARC0042944 and ALGA0014711) did not pass the call rate criterion (95%) but were included for further analysis in a sub-population of the samples (see below).

To further investigate the relationships of *CAST* with the regions detected on SSC2, four informative SNPs of *CAST* in the panel (ASGA0096522, ASGA0011047, MARC0042944 and ALGA0014711) and three other important markers in *CAST* associated with meat tenderness, including *Ser66Asn*, *Ser638Arg* and 41642_408i, were genotyped in a sub-population of the samples (n = 831) using a custom SNP panel [13]. The LD between SNP11 (SSC2_109) and the seven SNPs in *CAST* were estimated (Table 3). Almost no LD was observed between SNP11 and the SNPs in *CAST*, except for MARC0042944 (r^2^ = 0.33). A closer analysis of the SNPs within *CAST* found that MARC0042944 was in moderate LD (0.21–0.61) with *Ser66Asn*, ALGA0014711, ASGA0096522 and 41642_408i, but in very weak LD with *Ser638Arg* (r^2^ = 0.08) and ASGA0011047 (r^2^ = 0.06). The LD between two other interesting SNPs (2 and 25) on SSC2 and *CAST* was also very weak (r^2^ = 0.00–0.10, data was not shown).

**Table 3:** LD (r^2^) between SNP11 and gene *CAST*.

SNPs in *CAST*	Location	No.^#^	SNP11	MARC0042944	ASGA0096522	41642_408i	*Ser66Asn*	ALGA0014711	*Ser638Arg*
**MARC0042944**	Intron 3	519	0.33						
**ASGA0096522**	Intron 3	519–831	0.00	0.24					
**41642_408i**	Intron 5	519–1675	0.05	0.21	0.04				
***Ser66Asn***	Exon 8	831–1675	0.07	0.61	0.44	0.16			
**ALGA0014711**	Intron 20	173–928	0.00	0.38	0.36	0.18	0.06		
***Ser638Arg***	Exon 29	519–1978	0.00	0.08	0.16	0.33	0.27	0.01	
**ASGA0011047**	Intron 30	519–1672	0.00	0.06	0.16	0.33	0.25	0.06	0.95

^#^: the number of animals in sub-population used for LD estimations in the row.

In order to partition the *CAST* effect, MARC0042944 (the only marker of *CAST* in LD with SNP11) was fitted in the model simultaneously to re-estimate the effect of SNP11, SNP2, SNP25 and SNP7 (the most important marker in region SSC17_20, which will be discussed below) on PSF (Table 4). As expected, *CAST* was significant for PSF (*P < 0*.*01*) with allele B showing a significant additive effect of -1.05 ± 0.40N (*P < 0*.*05*). GWAS within population 2 also showed that MARC0042944 was the best marker (RSQ = 0.99) in region SSC2-106 associated with the trait (data was not shown). After accounting for the *CAST* effect, SNP11 was still significant for PSF (*P < 0*.*001*) with AA identified as the favorable genotype with a significant additive effect of -1.94 ± 0.79 N (*P < 0*.*01*). In addition, SNP11 showed a trend for an effect on meat Minolta colour (L*, a*, and b*) and crude protein content at *P* < 0.10 (S1 Table). SNP2 (*P < 0*.*001*) and SNP25 (*P < 0*.*01*), the other two important SNPs detected on SSC2, also remained in the model and showed a significant effect for PSF after accounting for *CAST* and SNP11. At the genome wide level, the revised GWAS in a sub-population which included all above six SNPs of *CAST* in the model showed that SSC2_5 and SSC2_109 were still the top two regions explained the largest genetic variance of the trait (data was not shown). All these findings indicated that the regions on SSC2 detected by GWAS had very weak relationships with *CAST*. Therefore, the effect of the detected regions on PSF might be distinct from, or interacting, with *CAST*.

**Table 4:** Significant effects (LSMs ± SE) of the important SNPs detected from GWAS and *CAST* (MARC0042944) on peak shear force (N) through multiple marker association.

SNP_Name	AA	AB	BB	Additive (B)
MARC0042944 (*CAST*)	45.49±0.80^a^ (501)	44.36±0.67^b^ (709)	43.38±0.80^b^ (198)	-1.05±0.40**
ASGA0008566 (SNP2)	43.26±0.88^A^ (199)	44.86±0.60^B^ (742)	45.11±0.67^B^ (467)	0.92±0.25*
ASGA0008695 (SNP25)	45.68±0.83^a^ (315)	44.81±0.65^a^ (707)	42.73±0.70^b^ (386)	-1.48±0.47**
DRGA0003285 (SNP11)	42.28±1.42^A^ (53)	44.79±0.59^B^ (452)	46.17±0.53^C^ (903)	1.94±0.79**
ASGA0075615 (SNP7)	45.56±0.62^A^ (885)	43.26±0.66^B^ (523)	n/a	n/a

Different subscript capital letters (A, B) among genotypes in each row means the difference at the significant level of *P < 0*.*001* and small letters means *P < 0*.*01*; For additive effect

** means significant level of *P < 0*.*01*

* means significant level of *P < 0*.*05;* The numbers in the parenthesis are the number of individuals for each genotype.

### Detailed analysis of region SSC17_20

Region SSC17_20 spans 965,447 base pairs from 20 to 21 Mb of the gene assembly and includes 18 SNPs on the 60K SNP panel. The 7^th^ SNP (SNP7, ASGA0075615) captured almost all of the GEBV variance of this region with near a perfect RSQ (0.966) (Table 2). A small haplotype block (S4 Fig) consisting of SNPs 2–6 showed little relationships with SNP7. SNP7 was considered as the most important marker representing this region in terms of an effect on PSF.

When considering the important markers on SSC2, very weak LD existed between SNP7 and SNPs on SSC2, including SNP2, SNP25, SNP11 and *CAST*. Therefore, the effect of region SSC17_20 on PSF is expected to be distinct from the regions detected on SSC2 and *CAST*. After accounting for the effect of *CAST* and SNPs on SSC2, SNP7 was still significant for PSF (*P < 0*.*001*), and BB was the favorable genotype with the lowest PSF (Table 4). Additional analysis found that SNP7 was also significant (*P < 0*.*05*) for other meat quality traits, such as drip loss (%), rib trim crude moisture content, fresh loin L* and a*, and fresh ham a* and b* (S1 Table).

### Candidate genes and their functional annotations

All the known genes in these detected regions are shown in Table 5. A total of 11 genes were identified in region SSC2_4. The most important SNP (2) in this region was intragenic (intron 1) in a novel Ensembl predicted gene (ENSSSCG00000021760) in pig. Region SSC2_5 was relatively gene rich including 43 known genes. The most important marker representing this region (SNP25) was located in exon 10 of *FRMD8* (FERM domain containing 8). For SNP26 and SNP20 which were in strong LD with SNP25, one was located in exon 1 of *SLC25A45* (solute carrier family 25, member 45) and the other was located in intron 17 of *LTBP3* (latent transforming growth factor beta binding protein 3). For regions SSC2_109 and SSC17_20, very few genes have been found in pig. The nearest gene to SNP11 and SNP7 were *FAM174A* and *PLCB4*, respectively.

**Table 5:** The candidate/nearest genes to the important SNPs in the detected regions.

Regions	Promising SNP	Ensembl Gene ID	Gene Start	Gene End	Gene name	Distance (bp)
SSC2_4	ASGA0008566 (SNP2)	ENSSSCG00000021760	4156989	4182781	Novel Ensembl prediction	Intron 3
	Other genes	*ADRBK1*, *KDM2A*, *RHOD*, *SYT12*, *PC*, *RCE1*, *C11orf80*, *CTSF*, *CCDC87*, *CCS*, *CTSF*, *KDM2A*				
SSC2_5	ASGA0008662 (SNP20)	ENSSSCG00000012985	5840778	5861803	*LTBP3*	Intron 17–18
	ASGA0008695 (SNP25)	ENSSSCG00000012992	5955700	5976369	*FRMD8*	Exon 10
	M1GA0002408 (SNP26)	ENSSSCG00000012993	5984015	5990284	*SLC25A45*	Exon 1
	Other genes	*PELI3*, *MRPL11*, *NPAS4*, *SLC29A2*, *B3GNT1*, *BRMS1*, *RIN1*, *CD248*, *TMEM151A*, *YIF1A*, *CNIH2*, *KLC2*, *PACS1*, *SF3B2*, *GAL3ST3*, *CATSPER1*, *CST6*, *CATSPER1*, *SART1*, *TSGA10IP*, *DRAP1*, *C11orf68*, *FOSL1*, *CCDC85B*, *FIBP*, *CTSW*, *EFEMP2*, *MUS81*, *CFL1*, *SNX32*, *OVOL1*, *AP5B1*, *KAT5*, *RNASEH2C*, *RELA*, *PCNXL3*, *TIGD3*, *DPF2*, *CDC42EP2*, *POLA2*				
SSC2_109	DRGA0003285 (SNP11)	ENSSSCG00000014178	109350241	109350513	*FAM174A*^*#*^	320,278
	Other genes	*CHD1*				
SSC17_20	ASGA0075615 (SNP7)	ENSSSCG00000007058	20571279	20770049	*PLCB4*	82,506
	Other genes	*LAMP5*, *PAK7*, *U6*				

^*#*^: unknown gene in *Sus scrofa*, the name in the table is the orthologue in other species.

Gene functional annotation (S2 Table) indicated that most of these genes were related to the maintenance of cellular components such as cell and organelle membranes, the extracellular and intracellular matrix, and cytoskeleton, all of which play important roles in maintaining the internal architecture of muscle cells. Further, GO terms involved demonstrated an enrichment of association with cell communication and energy transduction. For example, most genes were related to the signal transduction, functions of binding proteins, ATP and transforming growth factors, or were participating in protein kinase and transferase activities (S2 Table). These molecular functions and biological activities might play important roles in the regulation of muscular activity post-mortem, such as protein oxidation and phosphorylation, or protein turnover, and as a result impact meat tenderization post-mortem.

## Discussion

### Genomic regions for pork shear force

In this study, three 1Mb regions on SSC2 and one region on SSC17 were found to explain a relatively large proportion of genetic variance for pork peak shear force. Up to now, most of the QTL mapped for pork shear force were detected using linkage analysis, which covered large regions of the genome. In these previous studies SSC2 was identified as an important chromosome for meat tenderness as a large number of the QTL mapped to the central part of the chromosome within 40–79 cM [21–24]. A recent association mapping study further narrowed this range to two target genes, *MYOD1* (~ 44.4 Mb) and *UBL5* (~ 68.9 Mb), which showed a significant effect on meat tenderness and other quality traits, such as fat deposition, pH 1h post-mortem and meatiness in Italian Large White pigs [25]. Other regions identified for shear force on SSC2 were around 103 cM [21,22] and at the distal part [23,26]. The SSC2_109 region might overlap or be linked to the region at 103 cM [18,19] due to differences between *Sus scrofa* assemblies and maps. A recent GWAS in Landrace-Duroc-Yorkshire pigs detected four SNPs located on SSC1 (255 Mb), SSC2 (5 Mb and 74 Mb) and SSC9 (1 Mb) significantly affecting slice shear force [14]. The region SSC2_5 is close to μ-calpain (*CAPN1*) and was also identified in the present study which will be discussed later. On SSC17, four QTL associated with shear force have previously been mapped with peaks at 9 cM [24], 11.8 cM [27], 37.81 cM and 63.16 cM [28] whereas this study identified a region at 20cM to be important.

Previous work identified *CAST* as a candidate gene for meat tenderness [8]. In this study, the *CAST* region (SSC2_106) was also detected in population 2, and MARC0042944 located in intron 3 of *CAST* was considered the best marker in this region with an effect on the trait. In the combined data set, a region very close to *CAST* (SSC2_109) was significantly associated with PSF. Calpastatin is a specific inhibitor of the calpains, an enzyme family which is believed to be responsible for post-mortem tenderization of muscle through degradation of myofibrillar and associated proteins [29–31]. In recent years, several mutations in *CAST* have been found to significantly affect meat tenderness and other meat quality traits [18,19,32]. The most important markers in *CAST* for meat tenderness are the three missense mutations of *Ser66Asn*, *Arg249Lys* and *Ser638Arg* first reported by Ciobanu et al. [19]. Thus, *CAST* seems to be the most important gene on SSC2 to affect pork shear force. However, because none of these three mutations were included in the 60K SNP panel, the contribution of the SNPs/genes to the effect of these detected regions on the trait still remains unknown. Therefore, further investigations of the genomic architecture of this region and its impact on meat tenderness are encouraged.

### Effects of the detected regions on shear force

Five SNPs of *CAST* were included in the 60K SNP panel and all of them were located in introns. Only two of them (ASGA0096522 and ALGA0014711) qualified for the final GWAS here based on genotype filtering, and neither of them showed a significant effect on PSF. Instead region SSC2_109 explained a large proportion of genetic variance of PSF, and SNP11 (DRGA0003285) was considered the best marker, as it explained more than 99% of the genetic variance of this region. Sequence analysis found that SNP11 was about 2.5 Mb downstream of *CAST*. After accounting for the effects of *CAST* and other SNPs (2, 25 and 7), SNP11 was still significant for PSF (*P < 0*.*05*). Therefore, we hypothesized that the important effect of region SSC2_109 on shear force is due to SNP11 which might be distinct from the effect of *CAST*.

Further analysis showed that almost no LD existed between SNP11 and SNPs in *CAST*, including the two non-synonymous mutations (*Ser66Asn* and *Ser638Arg*). Ciobanu et al. [19] found that haplotypes of these mutations had a significant effect on meat tenderness, cooking loss, and juiciness. These two markers have been widely studied and their effects on meat tenderness has been validated in different breeds or populations [6,8,18,28,33–35]. A proteomic analysis also showed that samples carrying different genotypes of *CAST* had different autolyzed μ-calpain activity 24 h and 72 h post-mortem, which affected post-mortem activation time of calpain and resulted in lower shear force and drip loss [36,37]. One SNP (MARC0042944) located in intron 3 of *CAST* had moderate LD with SNP11, and showed an important effect on PSF in population 2. Although, no previous association of MARC0042944 on meat quality has been reported to date, in this study MARC0042944 was detected in population 2 and also showed a significant effect on peak shear force in the sub-population of samples tested. The remaining four SNPs in *CAST* were in very weak LD with SNP11. These findings indicated the possibility of other genes/markers in this region affecting shear force, which may be distinct from, or interacting, with *CAST*.

Two other regions (4 Mb and 5 Mb) on SSC2 also contributed a large effect on PSF. These two regions were more than 100 Mb away from *CAST* and no significant LD was observed between these regions and any SNPs in *CAST*. After accounting for the effect of *CAST* and SNP11 (SSC2_109), these two regions still had a significant effect on PSF. Region SSC2_4 has not previously been reported to be associated with QTL for shear force. A recent GWAS reported that one SNP (H3GA0005672) in region SSC2_5 was significantly associated with pork slice shear force [14], but it was not detected here. Instead three other SNPs (20, 25 and 26) in very strong LD were found to have significant associations with PSF. Further analysis showed that these three SNPs were in moderate LD (r^2^ = 0.32) with H3GA0005672. Region SSC2_5 is close to the gene μ-calpain (*CAPN1*) which was reported to affect beef and pork tenderness through interaction with *CAST* [38–40]. We also found evidence of a significant (*P < 0*.*05*) effect of these two regions on other meat quality traits, such as a* values of ham and loin (S1 Table). Analysis of the sequence also found that the important SNPs (2, 20, 25 and 26) in regions SSC2_4 and SSC2_5 were all intragenic with other known genes, which provided an opportunity to review the effect of these genes on meat tenderness (see next section).

The most important marker (SNP7) at 20 Mb on SSC17 had a significant (*P < 0*.*01*) effect on PSF. However, only two genotypes (AA and AB) of SNP7 were observed in this study (n = 1,976). Although four QTL have been mapped on SSC17 [24,27,28] by linkage mapping and association, none of them was found in the present study. A molecular genome scan identified a wide region corresponding to 54–64 Mb (assembly 10.0) on SSC17 associated with pork tenderness, pH and colour [26]. Many genes in this region were further confirmed to affect these meat quality traits [41]. However, this region was not detected in this study reported here. Instead, a 1 Mb region at 20 Mb on SSC17 was detected for PSF which has not been reported before. These findings provided an opportunity to investigate potential new genes and/or markers on SSC17 affecting meat tenderness. Furthermore, we also found evidence of effects of this region (SNP7) on other pork quality traits with *P < 0*.*05* (S1 Table).

### Possible new genes affecting peak shear force

A total of 56 known genes are found in the regions of SSC2_4 and SSC2_5 in the pig (http://uswest.ensembl.org/biomart/martview/). The best SNPs (2 and 25) along with two others in very strong LD with SNP25 were intragenic in *FRMD8*, *SLC25A45*, *LTBP3* and a novel gene (ENSSSCG00000021760) in pig. We assume that these genes or others close to these regions may play important roles on meat tenderization post-mortem.

*FRMD8* is a protein-coding gene with its product located mainly in the cytoskeleton which forms the internal framework of cells, and intracellular non-membrane-bounded organelles. There is limited functional study of *FRMD8*, however, an important paralogue of *FRMD8* is *KRIT1* (Ankyrin Repeat Containing) which plays a key role in the maintenance of intracellular reactive oxygen species (ROS) homeostasis so that oxidative cellular damage is prevented through antioxidant pathways. Oxidation is one of the important processes that cause extensive modifications encountered by the pig muscle proteome post-mortem, which is significantly associated with post-mortem events, such as tenderization [42,43]. Studies have demonstrated that the shift in energy metabolism after slaughter due to the lack of oxygen supply leads to the formation of ROS that cause oxidative damage to proteins [44]. It is possible that the effect of *FRMD8* on peak shear force might be related to its anti-oxidative activities that support maintenance of cytoskeleton integrity and intracellular ROS homeostasis and control the rate of apoptosis. A proteomics review of pork tenderization revealed that a sharp increase (57%) in oxidation levels in sarcoplasmic proteins takes place during the first four days after slaughter, and the oxidation levels of 70 myofibrillar proteins were observed to increase during a 48 h post-mortem period in meat with high water holding capacity [45]. Post-mortem proteolysis of myofibrillar and myofibrillar-associated proteins are the main determinants of meat tenderness [44].

SNP26 was in complete LD with SNP25 and was intragenic with *SLC25A45* which belongs to the SLC25 family of mitochondrial carrier proteins [46]. Functional study of this gene is very limited. An important paralog of this gene is member 20 in the same family (*SLC25A20*) which mediates the transport of acylcarnitines into the mitochondrial matrix for their oxidation by the mitochondrial fatty acid oxidation pathway, which might be related to the energy homeostasis of the muscle cell post-mortem and thus affecting the meat tenderization.

The protein encoded by *LTBP3* forms a complex with transforming growth factor beta (*TGFB*) proteins and plays critical roles in controlling and directing the activity of *TGFB1*. *TGFB1* encodes a multifunctional protein which is essential for function of many cell types in controlling proliferation, differentiation, adhesion and migration. *LTBP3* also plays a structural role in the extracellular matrix (ECM). The ECM is composed primarily of connective tissue [47], and many studies have revealed that connective tissue, as an integral component of muscle that transmits contractive force from the myofibrils in the postnatal animal, is a predominant contributor to meat toughness [44,48]. The present GWAS results revealed that one SNP (20) located in intron 17 of *LTBP3* showed an effect on peak shear force, which might be due to the structural role of *LTBP3* in ECM and the functional activities in the muscle cell through regulating *TGFB1*.

Additionally, it is noteworthy that SNP25 was about 173 Kb upstream from μ-calpain (*CAPN1*). This region was also found to be significant for pork slice shear force [14] (see above). Markers in *CAPN1* have been reported to influence the expression of the calpain-calpastatin enzyme complex, which regulates the rate of protein degradation in the live animals and post-mortem muscle [49], and consequently affects beef and pork tenderness [38–40]. However, two SNPs intragenic with *CAPN1* on the 60K SNP panel were not found to be associated with peak shear force in the present study.

The known gene information for SSC2_109 and SSC17_20 was very limited, which restricted further candidate gene analysis. SNP11 representing SSC2_109 was about ~320 Kb downstream of an unknown gene in pig, and the orthologue of this gene in other species is *FAM174A* (Family with sequence similarity 174, Member A), which also lacks functional information. CAST, about 2.5 Mb upstream to SNP11, was one of the few genes known to be responsible for meat tenderization post-mortem through its role in muscle protein degradation [18,19,30,32,45,50]. However, SNP11 seemed to be unrelated to CAST due to the very weak LD and little interactive effect on PSF. Therefore, there may be other genes in this region involved in meat tenderization. Re-sequencing this region in suitable animals may identify new candidate genes and causative mutations affecting pork quality. The most important marker (SNP7) in SSC17_20 was about 82 Kb upstream to *PLCB4* (Phospholipase C, Beta 4). Phospholipases are ubiquitously expressed and have diverse biological functions including roles in inflammation, cell growth, signaling and death and maintenance of membrane phospholipids. GO annotations related to *PLCB4* include phosphatidylinositol phospholipase C activity and calcium ion binding. These functions may also play important roles in meat tenderization post-mortem.

## Conclusions

Three 1 Mb genomic regions at 4 Mb, 5 Mb and 109 Mb on SSC2 and one region at 20 Mb on SSC17 have been found to explain a relatively large proportion of genetic variance for pork loin peak shear force. The SNPs of ASGA0008566, ASGA0008695, DRGA0003285 and ASGA0075615 were the four markers found to be most strongly associated with the effect and could be considered for marker assisted selection for meat tenderness improvement. Regions of SSC2_4 and SSC2_5 were enriched for known genes. The genes *FRMD8*, *SLC25A45* and *LTBP3* were potential candidate genes for association with meat tenderness, as they play important roles in muscle cell oxidative activities and the integrity of the ECM, which are two important determinants for meat tenderization post-mortem. The μ-calpain gene was also found close to this region but was not associated with PSF in this study. Few known genes have been mapped in regions SSC2_109 and SSC17_20. Very weak LD existed between SSC2_109 and *CAST* potentially indicating new genes affecting the trait in this region. Re-sequencing of these regions may help resolve the molecular architecture of the region. Study of the gene expression of these genes is encouraged to further identify the molecular mechanisms and potential causative mutations affecting this trait.

## Supporting Information

S1 Fig Haplotype block pattern (r^2^-scheme) for the region SSC2_4 based on the LD (r^2^) among the 11 SNPs within this region. The numbers on the top indicated the SNP order in this region; SNP in the green circle was the most important marker representing this region affecting the trait; The SNPs grouped in each triangle box indicated they were grouped in one block based on LD information.
(PNG)

S2 Fig Haplotype block pattern (r^2^-scheme) for the region SSC2_5 based on the LD (r^2^) among the 26 SNPs within this region. The numbers on the top indicated the SNP order in this region; SNP in the green circle was the most important marker representing this region affecting the trait; The SNPs grouped in each triangle box indicated they were grouped in one block based on LD information.
(PNG)

S3 Fig Haplotype block pattern (r^2^-scheme) for the region SSC2_109 based on the LD (r^2^) among the 17 SNPs within this region. The numbers on the top indicated the SNP order in this region; SNP in the green circle was the most important marker representing this region affecting the trait; The SNPs grouped in each triangle box indicated they were grouped in one block based on LD information.
(PNG)

S4 Fig Haplotype block pattern (r^2^-scheme) for the region SSC17_20 based on the LD (r^2^) among the 18 SNPs within this region. The numbers on the top indicated the SNP order in this region; SNP in the green circle was the most important marker representing this region affecting the trait; The SNPs grouped in each triangle box indicated they were grouped in one block based on LD information.
(PNG)

S1 Table Significant test (*P_value*) of the multiple markers on other meat quality traits. The full name of the SNPs in first row can be found in Table 2. “ns” means non-significant with P > 0.10.
(DOCX)

S2 Table Functional annotation of the candidate/nearest genes in the detected QTL. (DOCX)
