# Supplementary figures and images for: Genome Wide Association Studies (GWAS) Identify QTL on SSC2 and SSC17 Affecting Loin Peak Shear Force in Crossbred Commercial Pigs
Source: PLoS One. 2016 Feb 22;11(2):e0145082. doi: 10.1371/journal.pone.0145082 (PMC4763188; doi:10.1371/journal.pone.0145082)

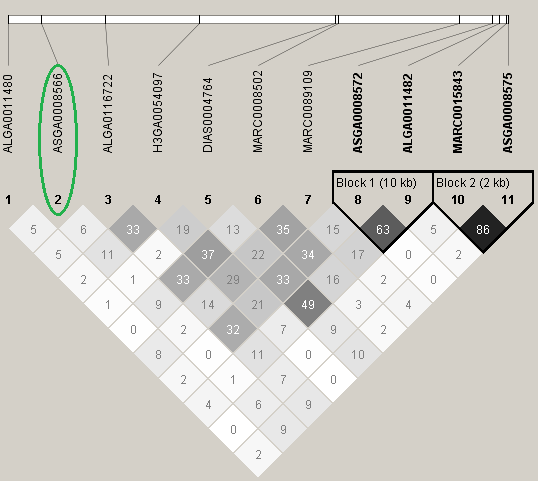

Supplement: S1 Fig — The numbers on the top indicated the SNP order in this region; SNP in the green circle was the most important marker representing this region affecting the trait; The SNPs grouped in each triangle box indicated they were grouped in one block based on LD information. (PNG) [file pone.0145082.s001.png]

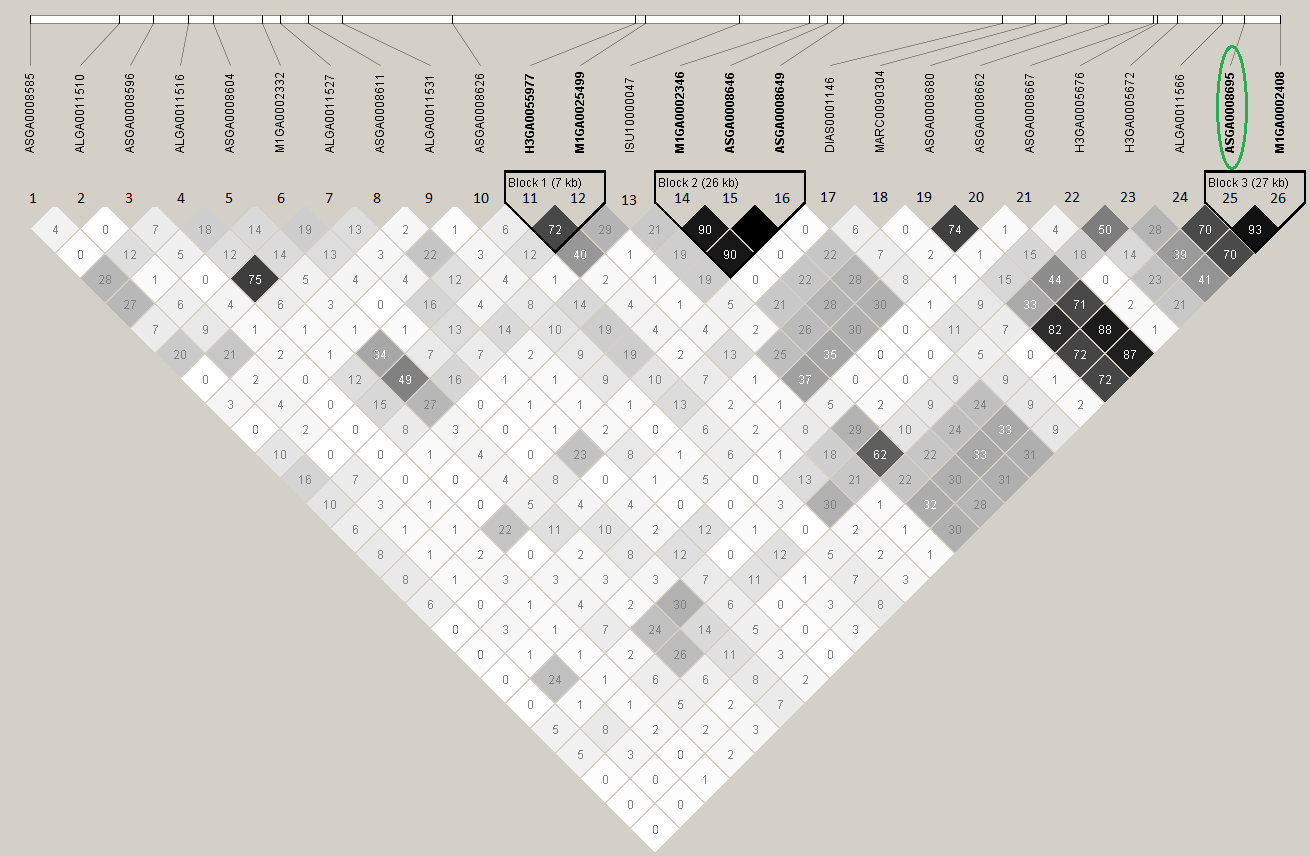

Supplement: S2 Fig — The numbers on the top indicated the SNP order in this region; SNP in the green circle was the most important marker representing this region affecting the trait; The SNPs grouped in each triangle box indicated they were grouped in one block based on LD information. (PNG) [file pone.0145082.s002.png]

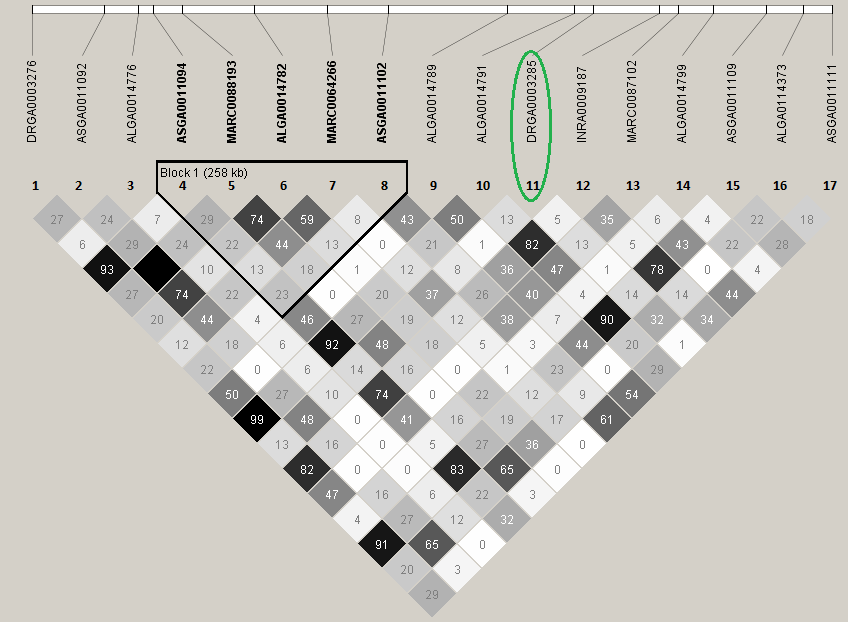

Supplement: S3 Fig — The numbers on the top indicated the SNP order in this region; SNP in the green circle was the most important marker representing this region affecting the trait; The SNPs grouped in each triangle box indicated they were grouped in one block based on LD information. (PNG) [file pone.0145082.s003.png]

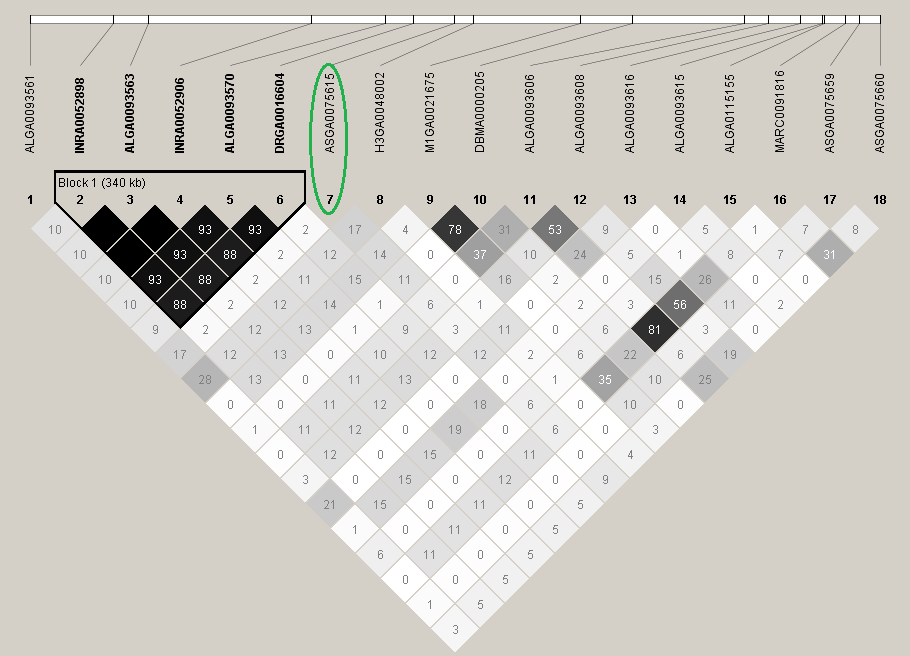

Supplement: S4 Fig — The numbers on the top indicated the SNP order in this region; SNP in the green circle was the most important marker representing this region affecting the trait; The SNPs grouped in each triangle box indicated they were grouped in one block based on LD information. (PNG) [file pone.0145082.s004.png]
