# Supplementary material for: Genome Wide Association Studies (GWAS) Identify QTL on SSC2 and SSC17 Affecting Loin Peak Shear Force in Crossbred Commercial Pigs
Source: PLoS One. 2016 Feb 22;11(2):e0145082. doi: 10.1371/journal.pone.0145082 (PMC4763188; doi:10.1371/journal.pone.0145082)
Supplement: S1 Table — The full name of the SNPs in first row can be found in Table 2. “ns” means non-significant with P > 0.10. (DOCX) [file pone.0145082.s005.docx]

**S1 Table. Significant test (*P_value*) of the multiple markers on other meat quality traits.**

| **Traits** | ***CAST^#^*** | **SNP2** | **SNP25** | **SNP11** | **SNP7** |
| --- | --- | --- | --- | --- | --- |
| Drip loss on fresh loin (%) | ns | ns | ns | ns | 0.012 |
| pH 24 h post mortem on fresh loin | 0.076 | ns | ns | ns | ns |
| Minolta L* on frozen/thawed loin | 0.074 | ns | ns | ns | ns |
| Minolta a* on frozen/thawed loin | ns | ns | 0.093 | ns | ns |
| Minolta b* on frozen/thawed loin | 0.033 | ns | ns | 0.080 | ns |
| Minolta L* on fresh loin | ns | ns | ns | ns | 0.048 |
| Minolta a* on fresh loin | ns | ns | ns | ns | 0.006 |
| Minolta b* on fresh loin | ns | ns | ns | 0.053 | ns |
| *subcutaneous fat* Minolta a* on fresh loin | ns | 0.024 | ns | ns | ns |
| *Gluteus medius* Minolta L* on fresh ham | 0.003 | ns | ns | 0.068 | ns |
| *Gluteus medius* Minolta a* on fresh ham | ns | ns | 0.006 | 0.021 | 0.001 |
| *Gluteus medius* Minolta b* on fresh ham | 0.035 | ns | ns | 0.001 | 0.059 |
| *Quadriceps femoris* Minolta L* on fresh ham | ns | ns | ns | ns | 0.075 |
| *Quadriceps femoris* Minolta a* on fresh ham | ns | ns | ns | 0.082 | ns |
| *Iliopsoas* Minolta L* on fresh ham | 0.004 | ns | ns | 0.067 | ns |
| *Iliopsoas* Minolta a* on fresh ham | ns | ns | ns | 0.062 | ns |
| Crude fat content (%) | 0.015 | ns | ns | ns | 0.084 |
| Crude protein content (%) | 0.005 | ns | ns | 0.082 | ns |
| Crude moisture content (%) | 0.009 | ns | ns | ns | 0.030 |

^#^: MARC0042944, in intron 3 of *CAST*;
