# Supplementary material for: Genome Wide Association Studies (GWAS) Identify QTL on SSC2 and SSC17 Affecting Loin Peak Shear Force in Crossbred Commercial Pigs
Source: PLoS One. 2016 Feb 22;11(2):e0145082. doi: 10.1371/journal.pone.0145082 (PMC4763188; doi:10.1371/journal.pone.0145082)
Supplement: S2 Table — (DOCX) [file pone.0145082.s006.docx]

**S2 Table. Functional annotation of the candidate/nearest genes in the detected QTL.**

| **Genes** | **Accession** | **Term** | **Category** |
| --- | --- | --- | --- |
| ***PLCB4*** | GO:0006629 | lipid metabolic process | BP_FAT |
|  | GO:0007165 | signal transduction | BP_FAT |
|  | GO:0016042 | lipid catabolic process | BP_FAT |
|  | GO:0035556 | intracellular signal transduction | BP_FAT |
|  | GO:0005790 | smooth endoplasmic reticulum | CC_FAT |
|  | GO:0014069 | postsynaptic density | CC_FAT |
|  | GO:0005634 | nucleus | CC_FAT |
|  | GO:0030425 | dendrite | CC_FAT |
|  | GO:0004435 | phosphatidylinositol phospholipase C activity | MF_FAT |
|  | GO:0004871 | signal transducer activity | MF_FAT |
|  | GO:0005509 | calcium ion binding | MF_FAT |
|  | GO:0005515 | protein binding | MF_FAT |
|  | GO:0008081 | phosphoric diester hydrolase activity | MF_FAT |
|  | GO:0016787 | hydrolase activity | MF_FAT |
| ***LTBP3*** | GO:0001501 | skeletal system development | BP_FAT |
|  | GO:0007179 | transforming growth factor beta receptor signaling pathway | BP_FAT |
|  | GO:0030502 | negative regulation of bone mineralization | BP_FAT |
|  | GO:0032331 | negative regulation of chondrocyte differentiation | BP_FAT |
|  | GO:0045780 | positive regulation of bone resorption | BP_FAT |
|  | GO:0046849 | bone remodeling | BP_FAT |
|  | GO:0060349 | bone morphogenesis | BP_FAT |
|  | GO:0060430 | lung saccule development | BP_FAT |
|  | GO:1902462 | positive regulation of mesenchymal stem cell proliferation/differentiation | BP_FAT |
|  | GO:0005576 | extracellular region/matrix/vesicular exosome | CC_FAT |
|  | GO:0005509 | calcium ion and protein binding | MF_FAT |
|  | GO:0050431 | transforming growth factor beta binding | MF_FAT |
| ***FRMD8*** | GO:0043232 | intracellular non-membrane-bounded organelle | CC_FAT |
|  | GO:0005856 | cytoskeleton | CC_FAT |
| ***SLC25A45*** | GO:0055085 | transmembrane transport | BP_FAT |
|  | GO:0005739 | mitochondrion | CC_FAT |
|  | GO:0016020 | membrane | CC_FAT |
|  | GO:0016021 | integral to membrane | CC_FAT |
| ***FAM174A*** | GO:0006414 | translational elongation | BP_FAT |
|  | GO:0005622 | intracellular | CC_FAT |
|  | GO:0005840 | ribosome | CC_FAT |
|  | GO:0003735 | structural constituent of ribosome | MF_FAT |
| ***CAST*** | GO:0030163 | protein catabolic process | BP_FAT |
|  | GO:2000675 | negative regulation of type B pancreatic cell apoptotic process | BP_FAT |
|  | GO:0010466 | negative regulation of peptidase activity | BP_FAT |
|  | GO:0010951 | negative regulation of endopeptidase activity | BP_FAT |
|  | GO:0005515 | protein binding | MF_FAT |
|  | GO:0044822 | poly(A) RNA binding | MF_FAT |
|  | GO:0030414 | peptidase inhibitor activity | MF_FAT |
|  | GO:0004869 | cysteine-type endopeptidase inhibitor activity | MF_FAT |
